# Ofloxacin induced leucopenia in complicated falciparum malaria: a case report

**DOI:** 10.4076/1757-1626-2-7097

**Published:** 2009-06-09

**Authors:** Swagata Tripathy, Amit Adhya

**Affiliations:** 1 Dept of Anaesthesia and Intensive Care, KIMSPatia, Bhubaneswar, OrissaIndia; 2 Dept of Pathology, KIMSPatia, Bhubaneswar, OrissaIndia

## Abstract

**Introduction:**

To report a case of ofloxacin-associated leucopoenia, which occurred in a patient of falciparum malaria shortly after administration and resolved following discontinuation of the drug.

**Case presentation:**

A 44 year old female was admitted in circulatory shock with a diagnosis of falciparum malaria, suffering from fever, diarrhoea and vomiting. After successful resuscitation she was treated with intravenous ofloxacin for Escherichia coli induced diarrhoea. She developed acute leucopoenia that resolved after discontinuation of the drug.

**Discussion:**

Ofloxacin is a broad-spectrum synthetic fluoroquinolone used for a wide variety of bacterial infections. Because of the temporal relationship between ofloxacin administration and the development of leucopenia in our patient, as well as the relationship between drug withdrawal and improvement in white blood cell count, ofloxacin-associated leucopenia was suspected. This reaction was categorized as probable according to the Naranjo probability scale. We report, for the first time in the English-language literature, a case of Ofloxacin-associated leucopoenia. This association is further supported by the exclusion of other potential causes for this adverse effect.

**Conclusion:**

Leucopoenia is a well-recognized adverse effect of several drugs. We report a case of Ofloxacin-associated leucopoenia during treatment of a patient with malaria. Healthcare personnel should be aware of this possible adverse reaction in patients treated with ofloxacin. A high degree of suspicion assumes special importance in this subgroup of critically ill patients with malaria where hematologic aberrations are common.

## Introduction

Drug induced leucopenia may complicate any clinical situation. We present a case of probable ofloxacin induced leucopenia in a patient with falciparum malaria where the condition reverted to normal promptly after stopping the drug. Cases of coexisting malaria and pancytopenia are not unknown in areas with a high prevalence of complicated falciparum malaria. A dilemma of deciding whether the leucopenia in a case of falciparum malaria is due to the disease process or an adverse drug reaction may arise at such times.

## Case presentation

A 44-year Asian-Indian female presented with fever, diarrhoea and vomiting of five days duration. She was diagnosed to have *Plasmodium falciparum* malaria elsewhere by immunochromatographic test. Prior treatment received was parenteral artesunate for three days. A worsening clinical status necessitated referral to our institute for management.

At presentation, the patient was in circulatory shock - heart rate 160/min, blood pressure 80/50mmHg. She was resuscitated with intravenous fluids and inotropic agents. Biochemical examination showed raised serum urea (58 mg/dl), creatinine (2.1 mg/dl), liver enzymes and normal electrolytes(Sodium 138 meq/dl, Potassium 4.2 meq/dl). Hematological parameters included normal leukocyte counts (8.6 × 109/l), mild anemia (Hemoglobin - 9.2 g/dl) and thrombocytopenia (platelets-150 × 109/l) (Table 1). Bleeding and clotting times were normal. Chest x ray was normal.

**Table 1.** Blood counts during the course of illness

DAY	Haemoglobin (g/dl)	TLC (× 10^9^/l)	DC (%)	Platelets (× 10^9^/l)	Reticulocyte (%)
**Ofloxacin started on DAY 3**					
Day 1	9.2	8.6	N57, E3, L39	150	0.3
DAY 3	10.0	8.2	N73, E2, L24	50	0.1
DAY 4	8.5	7.0	N72, E1, L25	50	0.1
DAY 5	7.3	1.6	N32, E6 L62	40	
**Ofloxacin stopped on DAY 6**					
DAY 6	10.2	1.5	N22, E6, L72	28	
DAY 7	10.3	2.7	N35, E2, L49	54	
DAY 9	9.6	10.0	N38, MMC2, MC9	81	5
Day 10	10.0	15.0	MC15*MC_+_St6*, E1, L32	150	6

Abbreviations: TLC: total leucocyte count; DC: differential count; N: neutrophils; E: eosinophils; L: lymphocytes; M: monocytes; MC: myelocytes; MMC: metamyelocyte; St: stab form.

Over the next two days signs of clinical deterioration suggestive of acute renal failure were noted. The patient continued to have severe diarrhoea. Alterations in haematological parameters showed worsening anemia (Hemoglobin -8 g/dl) and thrombocytopenia (50 × 109/l). Total leucocyte counts were stable (8.2 × 109/l). At this stage stool culture grew colonies of *Escherichia coli* which were sensitive to ofloxacin and carbapenem. The patient was started on intravenous ofloxacin (200 mg twice a day) after adjusting for impaired renal function.

After five days of treatment patient continued to deteriorate. She showed persistent oozing from site of puncture. Altered hematological profile included a drop in platelet counts, hemoglobin and packed cell volume. Severe leucopenia (1.6 × 109/l) was noted.

Peripheral blood smear and quantitative buffy coat test for malaria parasite were negative. Erythrocyte sedimentation rate was 45, 55 and 50 mm in first hour and C reactive protein of 65, 68 and 61 mg/dl on first, fourth and sixth days respectively. Chest X ray did not show any evidence of new infection. Ultrasonography of abdomen and pelvis was normal. Microbiological cultures form blood, tracheal secretions and urine failed to show any growth. Serial tests of sputum were negative for acid fast bacilli. Widal test was non reactive. On day six, hematologic profile continued to deteriorate (Table1).

Drug induced leucopenia was the provisional diagnosis. The patient was on injection ofloxacin, artesunate, pre and probiotics for diarrhoea, rabeprazole, salbutamol and dopamine infusion. Ofloxacin was discontinued at this stage.

**Figure 1.**
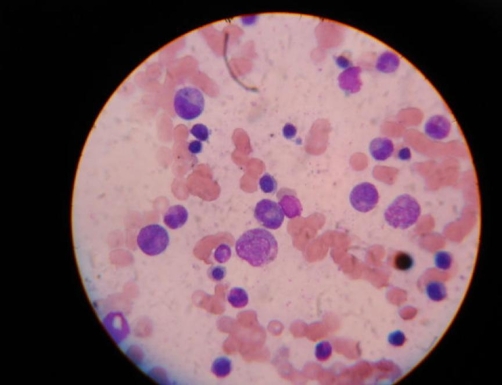
Bone marrow aspirate showing maturation arrest in the WBC series and erythroid hyperplasia. (leishman stain, ×100).

Hematological profile showed signs of stabilization on day seven. The cell counts started improving (Table 1) and reached near normal levels by day ten of treatment. The patient had received no blood or component transfusion in the interim. Bone marrow done on day eight showed hypercellularity and signs of regeneration (Figure 1). Bone marrow differential counts revealed an increased promyelocye (26%) and myelocyte count (18%). (Table 2)

**Table 2.** Bone marrow differential counts on eighth day

	Differential (%)	
Cell type	Case	Normal range (%)
Myeloblast	2	0.3-5.0
Promyelocyte	26	1-8
Myelocyte	18	8-16
Metamyelocyte + stab form	4	18-40
Segmented neutrophill	30	3-11
Eosinophil	1	1-5
Normoblast	18	18-36
Lymphocyte	17	11-23
Mononcyte	1	0-0.8
Plasma cell	1	0.4-3.9
Myeloid:erythroid ratio	1.1	1.5-3.3

**Figure 2.**
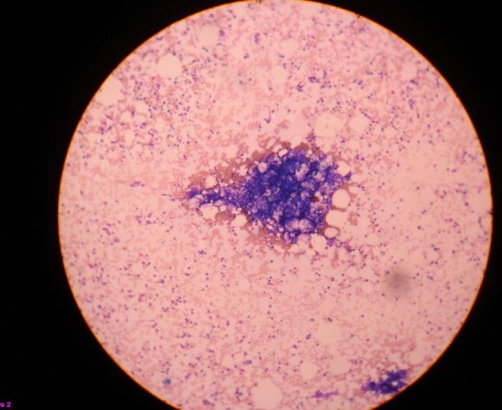
Bone marrow aspirate showing a marrow particle which is hypercellular. (leishman stain, ×10).

She developed late-onset ventilator associated pneumonia by 18^th^ day and was put on Carbapenem therapy. She stabilized with residual renal compromise requiring haemodialysis and was discharged after one month with complete recovery.

## Discussion

Leucopenia in a critically ill patient can be attributed to various causes. Falciparum malaria is associated with anemia and thrombocytopenia due to hemophagocytosis and hemolysis. Leucopenia and pancytopenia are less common [1-3]. Lathia and colleagues have shown that thrombocytopenia alone (platelet count less than 150,000 mm^-3^) was a predictor for malaria and in combination with anemia (Hb < 10 g/dl) it was next best parameter. RDW and leukocyte count were not predictive [4]. Our patient had received a full course of antimalarial treatment and had no evidence for presence of parasites at the time of the episode of sudden leucopenia.

Other infections (tuberculosis, salmonellosis etc), sepsis and connective tissue diseases can cause macrophage activation syndrome [5] and leucopenia. Repeated smears showed absolute pancytopenia with no evidence of premature cells or hemolysis. Bone marrow biopsy did not reveal hemophagocytosis; splenomegaly was not documented. There was no evidence of severe sepsis in our patient in the form of rising CRP levels, or positive microbial cultures.

Drug-induced leukopenias can occur in a dose-dependent relationship or in an idiosyncratic, dose-independent hypersensitivity reaction. The antimalarial drug artesunate has proven to be very safe and the only incidents of hematologic adverse effects associated with it occur when it is used in combination therapy [6] - with amodiaquine an agent known to cause leucopenia [7]. None of the other drugs being administered to our patient are known to cause this adverse effect. No drug other than Ofloxacin was discontinued prior to the rapid improvement in leucocyte counts.

Ofloxacin is a commonly used fluoroquinolone agent in gastrointestinal infections and been associated with adverse effects such as seizures, hypersensitivity vasculitis, hemoglobinurea etc [8,9].

Other drugs of this group have been implicated in serious hematologic adverse effects. Search of literature led us to similar cases where ciprofloxacin, levofloxacin, trovafloxacin and moxifloxacin were implicated as the cause of neutropenia, pancytopenia or bone marrow suppression [10-13]. The exact mechanism of action of these reactions are not well known till date. Having been administered for two days in a modified dose, serum level was not investigated. Re-challenge with the drug was not done due to ethical reasons.

The temporal sequence of events implicates ofloxacin as a probable cause of bone marrow suppression Naranjo probability scale score 6 [14] which exacerbated the pre-existing anaemia and thrombocytopenia. An idiosyncratic reaction cannot be ruled out.

## Conclusion

Leucopenia in falciparum malaria is less common than anemia and thrombocytopenia.

- Sudden onset leucopenia is more likely to be drug related.
- Ofloxacin may cause leucopenia as reported for other Quinolones.
- Suspected/most relevant drug possibly causing the leucopenia should be promptly stopped.
